# Supplementary material for: A cell fate decision map reveals abundant direct neurogenesis bypassing intermediate progenitors in the human developing neocortex
Source: Nat Cell Biol. 2024 Mar 28;26(5):698–709. doi: 10.1038/s41556-024-01393-z (PMC11098750; doi:10.1038/s41556-024-01393-z)
Supplement: Supplementary file 2 — Reporting Summary [file 41556_2024_1393_MOESM2_ESM.pdf]

nature portfolio

Corresponding author(s): Alexandre BAFFET

Last updated by author(s): 22/08/2023

Reporting Summary

Nature Portfolio wishes to improve the reproducibility of the work that we publish. This form provides structure for consistency and transparency in reporting. For further information on Nature Portfolio policies, see our Editorial Policies and the Editorial Policy Checklist.

Statistics

For all statistical analyses, confirm that the following items are present in the figure legend, table legend, main text, or Methods section.

n/a	Confirmed	
☐	☒	The exact sample size (*n*) for each experimental group/condition, given as a discrete number and unit of measurement
☐	☒	A statement on whether measurements were taken from distinct samples or whether the same sample was measured repeatedly
☐	☒	The statistical test(s) used AND whether they are one- or two-sided<br>*Only common tests should be described solely by name; describe more complex techniques in the Methods section.*
☒	☐	A description of all covariates tested
☒	☐	A description of any assumptions or corrections, such as tests of normality and adjustment for multiple comparisons
☐	☒	A full description of the statistical parameters including central tendency (e.g. means) or other basic estimates (e.g. regression coefficient) AND variation (e.g. standard deviation) or associated estimates of uncertainty (e.g. confidence intervals)
☐	☒	For null hypothesis testing, the test statistic (e.g. *F*, *t*, *r*) with confidence intervals, effect sizes, degrees of freedom and *P* value noted<br>*Give P values as exact values whenever suitable.*
☒	☐	For Bayesian analysis, information on the choice of priors and Markov chain Monte Carlo settings
☒	☐	For hierarchical and complex designs, identification of the appropriate level for tests and full reporting of outcomes
☒	☐	Estimates of effect sizes (e.g. Cohen's *d*, Pearson's *r*), indicating how they were calculated

Our web collection on statistics for biologists contains articles on many of the points above.

Software and code

Policy information about availability of computer code

Data collection	Metamorph 7.10
Data analysis	Fiji, Matlab R2023B and Prism 9. The LiveFixedCorrelative code can be downloaded at https://xfer.curie.fr/get/mBUYU6SjQ6T/LiveFixedCorrelative%20Code.zip

For manuscripts utilizing custom algorithms or software that are central to the research but not yet described in published literature, software must be made available to editors and reviewers. We strongly encourage code deposition in a community repository (e.g. GitHub). See the Nature Portfolio guidelines for submitting code & software for further information.

Data

Policy information about availability of data

All manuscripts must include a data availability statement. This statement should provide the following information, where applicable:

- Accession codes, unique identifiers, or web links for publicly available datasets
- A description of any restrictions on data availability
- For clinical datasets or third party data, please ensure that the statement adheres to our policy

The live imaging and immunofluorescence data that support the findings of this study are available from the corresponding author (alexandre.baffet@curie.fr) or from the first author (coquand.laure@gmail.com) upon request. The LiveFixedCorrelative code is available at https://xfer.curie.fr/get/mBUYU6SjQ6T/LiveFixedCorrelative%20Code.zip

March 2021

Human research participants

Policy information about studies involving human research participants and Sex and Gender in Research.

Reporting on sex and gender	Sex and Gender have not been taken into account , as this was out of the scope of this study
Population characteristics	N/A (anonymzed post-mortem fetal samples)
Recruitment	N/A
Ethics oversight	French biomedical agency (Agence de la Biomédecine, approval number: PFS17-003)

Note that full information on the approval of the study protocol must also be provided in the manuscript.

Field-specific reporting

Please select the one below that is the best fit for your research. If you are not sure, read the appropriate sections before making your selection.

☒ Life sciences ☐ Behavioural & social sciences ☐ Ecological, evolutionary & environmental sciences

For a reference copy of the document with all sections, see nature.com/documents/nr-reporting-summary-flat.pdf

Life sciences study design

All studies must disclose on these points even when the disclosure is negative.

Sample size	Due to the very complex nature of this live imaging-based method, sample size was limited to 3 replica (and sometimes 2 for human fetal tissues, depending on its availability).
Data exclusions	No data was excluded from the analysis
Replication	All attempts for replication were successful. Experiments were replicated at least 3 times or twice for human fetal tissue samples
Randomization	There was no randomization, as we did not compare conditions in this study, but rather quantified different behaviors within control samples.
Blinding	no blinding applied as all experiments were performs in the same control samples.

Reporting for specific materials, systems and methods

We require information from authors about some types of materials, experimental systems and methods used in many studies. Here, indicate whether each material, system or method listed is relevant to your study. If you are not sure if a list item applies to your research, read the appropriate section before selecting a response.

Materials & experimental systems

n/a	Involved in the study	
☐	☒	Antibodies
☐	☒	Eukaryotic cell lines
☒	☐	Palaeontology and archaeology
☒	☐	Animals and other organisms
☒	☐	Clinical data
☒	☐	Dual use research of concern

Methods

n/a	Involved in the study	
☒	☐	ChIP-seq
☒	☐	Flow cytometry
☒	☐	MRI-based neuroimaging

Antibodies

Antibodies used	Antibodies used in this study were mouse anti-SOX2 (Abcam Ab79351, clone 9-9-3, 1/500), sheep anti-EOMES (R&D Sytems AF6166, 1/500), rabbit anti-NEUN (Abcam Ab177487, 1/500), chicken anti-GFP (Abcam Ab13970, 1/500), mouse anti-pVimentin (Abcam Ab22651, clone 4A4, 1/1000), rat anti-HES1 (MBL D134-3, clone NM1, 1/500), rabbit anti-NeuroD2 (Abcam, ab104430, 1/500), mouse anti-HuC/HuD (ThermoFisher Scientific, A-21271, clone 16A11, 1/200), rabbit anti-HOPX (Proteintech, 11419-1-AP, 1/500), mouse anti-S100B (Synaptic systems 287111, clone 86D7E4, 1/500), mouse anti-OLIG2 (Millipore MABN50, clone 211F1.1, 1/200), mouse anti-LIFR (Abcam 89792, clone MM0455-9B23, 1/50), rabbit anti-PTPRZ1 (Sigma HPA015103 (Atlas antibodies), 1/500). Secondary antibodies used were: Donkey Anti-Sheep IgG H&L (Alexa Fluor® 405) Abcam ab175676; DyLight™ 405 AffiniPure™ Donkey

Anti-Mouse IgG (H+L) Jackson ImmunoResearch 715-475-150; DyLight™ 405 AffiniPure™ Donkey Anti-Rabbit IgG (H+L) Jackson ImmunoResearch 711-475-152; Alexa Fluor® 488 AffiniPure™ Donkey Anti-Rabbit IgG (H+L) Jackson ImmunoResearch 711-545-152; Donkey anti-Rabbit IgG (H+L) Highly Cross-Adsorbed Secondary Antibody, Alexa Fluor™ Plus 488 Thermo Fisher A32790; Donkey anti-Chicken IgY (H+L) Highly Cross Adsorbed Secondary Antibody, Alexa Fluor™ 488 Thermo Fisher A78948; Alexa Fluor® 488 AffiniPure™ Donkey Anti-Chicken IgY (IgG) (H+L) Jackson ImmunoResearch 703-545-155; Alexa Fluor® 488 AffiniPure™ Donkey Anti-Mouse IgG (H+L) Jackson ImmunoResearch 715-545-150; Donkey anti-Mouse IgG (H+L) Highly Cross-Adsorbed Secondary Antibody, Alexa Fluor™ Plus 488 Thermo Fisher A32766; Donkey anti-Sheep IgG (H+L) Cross-Adsorbed Secondary Antibody, Alexa Fluor™ 568 Thermo Fisher A21099; Cy™3 AffiniPure™ Donkey Anti-Sheep IgG (H+L) Jackson ImmunoResearch 713-165-147; Donkey anti-Mouse IgG (H+L) Highly Cross-Adsorbed Secondary Antibody, Alexa Fluor™ 568 Thermo Fisher A10037; Cy™3 AffiniPure™ Donkey Anti-Mouse IgG (H+L) Jackson ImmunoResearch 715-165-150; Donkey anti-Rabbit IgG (H+L) Highly Cross-Adsorbed Secondary Antibody, Alexa Fluor™ 568 Thermo Fisher A10042; Cy™3 AffiniPure™ Donkey Anti-Rabbit IgG (H+L) Jackson ImmunoResearch 711-165-152; Donkey anti-Rabbit IgG (H+L) Highly Cross-Adsorbed Secondary Antibody, Alexa Fluor™ Plus 647 Thermo Fisher A32795; Alexa Fluor® 647 AffiniPure™ Donkey Anti-Rabbit IgG (H+L) Jackson ImmunoResearch 715-605-152; Donkey anti-Goat IgG (H+L) Highly Cross-Adsorbed Secondary Antibody, Alexa Fluor™ Plus 647 Thermo Fisher A32849; Alexa Fluor® 647 AffiniPure™ Donkey Anti-Goat IgG (H+L) Jackson ImmunoResearch 705-605-003; Alexa Fluor® 647 AffiniPure™ Donkey Anti-Mouse IgG (H+L) Jackson ImmunoResearch 715-605-150.

Validation

We validated these commonly-used antibodies, based on localization, expression patterns, and co-localization with other overlapping cell fate markers.
-mouse anti-SOX2 (Abcam Ab79351) manufacturer statement: Suitable for: ICC/IF, WB, Flow Cyt (Intra). Reacts with: Mouse, Human
-Sheep anti-EOMES (R&D Sytems AF6166) manufacturer statement: Detects human EOMES in Western blots
-rabbit anti-NEUN (Abcam Ab177487) manufacturer statement: Suitable for: Flow Cyt (Intra), IHC (PFA fixed), mIHC, IHC-P, WB, ICC/IF, IHC-Fr. Reacts with: Mouse, Rat, Sheep, Goat, Cat, Dog, Human, Zebrafish, Common marmoset
-chicken anti-GFP (Abcam Ab13970) manufacturer statement: Suitable for: WB, ICC/IF. Reacts with: Species independent
-mouse anti-pVimentin (Abcam Ab22651). manufacturer statement: Suitable for: ICC/IF, WB, Flow Cyt (Intra). Reacts with: Mouse, Human
-rat anti-HES1 (MBL D134-3) manufacturer statement: Application: ICC, IHC, IP, WB
-rabbit anti-NeuroD2 (Abcam, ab104430). manufacturer statement: Suitable for: IHC-P, IHC-Fr, WB. Reacts with: Mouse, Human
-mouse anti-HuC/HuD (ThermoFisher Scientific, A-21271). manufacturer statement: Immunocytochemistry (ICC/IF). Published species Avian, Cat, Chicken, Chimpanzee, Fish, Guinea pig, Horse, Human, Lizard, Mouse, Non-human primate, Pig, Rabbit, Rat, Reptile, Rhesus monkey, Rodent, Shark, Sheep, Xenopus, Zebrafish
-rabbit anti-HOPX (Proteintech, 11419-1-AP). manufacturer statement: Published Applications: IF; Tested Reactivity Human, Mouse, Rat
-mouse anti-S100B (Synaptic systems 287111). manufacturer statement: Applications: IF.
-mouse anti-OLIG2 (Millipore MABN50) manufacturer statement: immunohistochemistry: suitable
-mouse anti-LIFR (Abcam 89792) manufacturer statement: Reacts with: Human
-rabbit anti-PTPRZ1 (Sigma HPA015103) manufacturer statement: species reactivity
human

Eukaryotic cell lines

Policy information about cell lines and Sex and Gender in Research

Cell line source(s)

- The feeder-independent iPS cell line used for this study was a gift from Silvia Cappello (Max-Plank Institute of Psychiatry - Munich). Cells were reprogrammed from NuFF3-RQ human newborn foreskin feeder fibroblasts (GSC-3404, GlobalStel)
The HEK-Phoenix-GP cell line was obtained from ATCC (CRL-3215)

Authentication

The iPSC line used in this study was genotyped. The HEK-Phoenix-GP cell line was authenticated by ATCC.

Mycoplasma contamination

Mycoplasma testing were performed weekly and always tested negatively.

Commonly misidentified lines
(See ICLAC register)

No commonly misidentified lines were used in this study.
